# Image Quality Assessment for Realistic Zoom Photos

**DOI:** 10.3390/s23104724

**Published:** 2023-05-13

**Authors:** Zongxi Han, Yutao Liu, Rong Xie, Guangtao Zhai

**Affiliations:** 1 Institute of Image Communication and Information Processing, Shanghai Jiao Tong University, Shanghai 200240, China; zongxihan@sjtu.edu.cn (Z.H.); xierong@sjtu.edu.cn (R.X.); 2 School of Computer Science and Technology, Ocean University of China, Qingdao 266100, China; liuyutao@ouc.edu.cn

**Keywords:** mobile imaging sensor, realistic zoom photos, over-sharpening effect, zoom quality metric, image sharpness, image naturalness

## Abstract

New CMOS imaging sensor (CIS) techniques in smartphones have helped user-generated content dominate our lives over traditional DSLRs. However, tiny sensor sizes and fixed focal lengths also lead to more grainy details, especially for zoom photos. Moreover, multi-frame stacking and post-sharpening algorithms would produce zigzag textures and over-sharpened appearances, for which traditional image-quality metrics may over-estimate. To solve this problem, a real-world zoom photo database is first constructed in this paper, which includes 900 tele-photos from 20 different mobile sensors and ISPs. Then we propose a novel no-reference zoom quality metric which incorporates the traditional estimation of sharpness and the concept of image naturalness. More specifically, for the measurement of image sharpness, we are the first to combine the total energy of the predicted gradient image with the entropy of the residual term under the framework of free-energy theory. To further compensate for the influence of over-sharpening effect and other artifacts, a set of model parameters of mean subtracted contrast normalized (MSCN) coefficients are utilized as the natural statistics representatives. Finally, these two measures are combined linearly. Experimental results on the zoom photo database demonstrate that our quality metric can achieve SROCC and PLCC over 0.91, while the performance of single sharpness or naturalness index is around 0.85. Moreover, compared with the best tested general-purpose and sharpness models, our zoom metric outperforms them by 0.072 and 0.064 in SROCC, respectively.

## 1. Introduction

With the rapid development of mobile CMOS imaging sensor techniques such as 2-layer transistor pixel and dual vertical gates [1,2,3,4,5], smartphone picture quality has been improved a lot and user-generated images and videos have become the mainstream of social media and our entertainment. Among these contents, zoom photos account for a large portion due to its ability to highlight subjects and make composition easy. For professional photographers, zoom capability (or focal length choices) with its shadow depth of field is one of determining factors in taking great pictures; while for average consumers, magnifying image without losing clarity is also very meaningful. But how to assess the realistic zoom image quality remains uncovered.

### 1.1. Difference between Zoom Photos and Gaussian Blur Images

**Figure 1:**
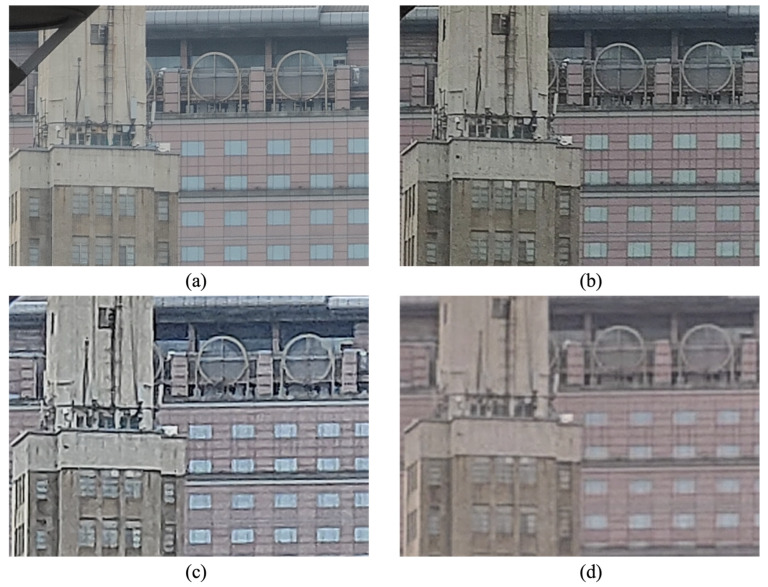
Four zoom photos with (**a**) high-quality details, (**b**) over-sharpening effect, (**c**) unnatural details, (**d**) ringing.

Although many phones have been equipped with one or more zoom lenses, their focal lengths are fixed and digital zoom is still the universal ways of magnifying images. The straightforward implementation is to crop a small portion of image, then interpolate pixel values by the nearest neighbor, bilinear or bicubic methods [6], but it would lead to pixelated or blurry details. To alleviate blurry appearances, some phone-makers post-process images through unsharp masking. Other manufacturers utilize learning-based super-resolution [7] or multi-frame stacking algorithms in order to add fine details in the delicate areas such as cement roads and rocks. Representative techniques are the deep fusion in iPhone and the super res zoom in Google Pixel [8]. However, both post-processing and synthesis-based approaches would produce over-sharpening effects, unnatural details and ringing into the image, for which traditional sharpness metrics over-estimate. In fact, this is where zoom photos differ from purely gaussian blurred images. To better visualize these artifacts, Figure 1b–d show the over-sharpening effect, unnatural details and ringing artifacts, respectively. It can be seen that, the details of Figure 1b look more grainy and noisier than Figure 1a because of over-sharpening; the lines of the buildings, windows, walls in Figure 1c are zigzag; and the ringing phenomenon appears around the edges of Figure 1d due to JP2K compression and over-sharpening. To our best of knowledge, this paper is the first attempt to deal with realistic zoom photos in the task of IQA.

### 1.2. Laboratory Measurements of Targets vs. Perceptual Metrics on Natural Photos

In industry, the edge blur is often measured through SFR or MTF, which can be calculated via a slant checkerboard, or the more complex Sine Siemens Star and Log-F contrast target. To overcome the texture loss problem due to edge-preserving filters, DxO Lab proposed the dead leaves model in [9], and the IE group came up with the Gaussian white noise target for the same purpose [10]. Although the correlation between measurements of both targets and subjective scores of photographic images has been confirmed in [11], there is no doubt that human-made targets are not as realistic and complex as natural photos. The measurement of the energy fall-off by DxO or the kurtosis changes of Gaussian noise image by IE are also more simpler than what current objective IQA algorithms model. Moreover, the over-sharpening phenomenon, which affects both edges and textures of the images, can’t be handled by these texture-blur measures. Therefore, in this paper, we address the zoom photo quality assessment problem realistically, instead of using laboratory targets and environments.

### 1.3. Limitations of Sharpness, Super-Resolution, and General-Purpose Metrics

Since the sharpness/blurriness is the most related quality factor for realistic zoom images and can be used to assess the zoom quality naturally, we review them next in more details.

In [12], the author first detected edge points by Sobel operator, then measured the sharpness through the edge width. This method works well for images with the same content, but can’t handle heterogeneous contents. Ferzli et al. [13] proposed the probability of just noticeable blur (JNB) in consideration of contrast masking effect. The edge width and local contrast were first computed in edge blocks, and then fed into a probabilistic summation model to estimate the whole image blur. To deal with images with different portions of background blur, a saliency map was incorporated into JNB [14]. Furthermore, by utilizing the cumulative probability, the same pooling effect is achieved under a unified framework [15]. Inspired by the triangle model of gradient profile, Yan et al. [16] defined the edge sharpness as the triangle height divided by the width. Vu et al. [17] combined the slope of spectrum magnitude with the total variation of pixel values in the spatial domain into a sharpness value S3. The spectral term is responsible for fine-detailed textures while the spatial part accounts for high-contrast edges. Thereafter, the same author proposed a fast sharpness index called FISH where the energy of different high-frequency sub-bands are accumulated in the wavelet domain [18]. Observing that different scale local phase maps align in strong image structures, Hassen et al. [19] measured the image sharpness through the local phase coherence. Besides image blur, LPC can respond effectively to noise contamination, which distinguishes itself from other sharpness metrics. Based on the re-blurring process, the sharpness was defined as the decreased percentage of fourth-order moments of the re-blurred version relative to its test image [20]. More recently, Li et al. [21] decomposed gradient image blocks into a novel set of Tchebichef basis. The square Tchebichef moments were normalized by the variance to compensate influence of image contents, and further weighted by a saliency model. Besides spatial and spectral domain, there are a few metrics making use of high-level features. Gu et al. [22] proposed a sharpness metric where autoregressive (AR) parameters are estimated, then the differential energy and contrast between AR values are linearly combined. Li et al. [23] and Han et al. [24] both measured the blurriness through sparse representation. The latter needs partial information of the original image, thus belonging to the reduced-reference category. Liu et al. [25] developed a new sharpness metric for realistic out-of-focus images where phase congruency and gradient magnitude maps are merged by max pooling, and further weighted through a saliency map to form the sharpness value.

Despite the success of sharpness index on gaussian blurred images, they generally over-estimate the sharpened details and artifacts in zoom photos. In [26,27], this over-shoot problem has been raised, but still limited to the simulated scenario. Moreover, the S3-III [27] performs moderately in our database. Another relevant work is the quality assessment for super-resolution [28,29,30]. These metrics often assume the existence of “original low-resolution image”. In contrast, our zoom quality metric only relies on the tested zoom photos and is thus no-reference. The distortion types are also more subtle and complex. It may be argued that general-purpose IQA metrics [31,32,33,34,35] can assess these artifacts correctly, but we will demonstrate that they are not effective as the sharpness metric for zoom photos in Section 4.

### 1.4. The Contribution of This Article

To tackle the above problem, we propose a novel zoom quality metric which incorporates the image sharpness and naturalness. For zoom photos, sharpness is the determining quality factor. To estimate it, we resort to the free energy theory which models the brain interpretation process by decomposing an image (scene) into the predicted and residual version [36]. Specifically, the gradient image is first encoded by sparse representation. Then the square sparse coefficients are summed for the reconstructed gradient image, and the entropy is computed for the residual part, respectively. The sharper a zoom photo is, the higher energy/entropy the reconstructed/residual image has. Thus, we form the sharpness index by adding these two quantities. As mentioned above, zoom photo differentiate itself from gaussian blurred image in the over-sharpening details and unnatural looks. To compensate such perceptually harmful effects, a set of parameters of MSCN distribution are first extracted from zoom photos, and then compared with that of natural images. Finally, this NSS score is combined with the sharpness index linearly. **In summary, our contributions are as follows:**

- First, we construct a realistic zoom database by collecting 20 phone cameras and shooting them in 45 texture-complex scenes. Mean expert opinion scores and several analyses are also provided. Compared to existing gaussian blur databases, our database contains the most authentically distorted photos and scenes. The image resolution is also the highest.
- We are the first to derive the whole formulation of the free-energy under the sparse representation model, according to which a new sharpness index is proposed.
- A novel zoom quality metric is proposed which incorporates image sharpness and naturalness. Natural scene statistics are included as a mechanism to prevent over-sharpening and penalize unnatural appearances.
- The zoom quality metric is tested in both unsharp masking simulations and the zoom photo database, which outperforms existing sharpness and general-purpose QA models.

The rest of this paper is organized as follows: Section 2 describes the construction of a realistic zoom image database. Section 3 explains two components of our sharpness metric and the important NSS score. Extensive experimental results are given in Section 4. Finally, Section 5 concludes the paper.

## 2. Realistic Zoom Photo Database

### 2.1. The Construction Process and Comparison with Other Databases

Zoom photo refers to the image shot at focal lengths several times longer than the main camera (e.g., 23–28 mm), which includes both optically and digitally zoomed ones. For their quality differences, there are three cases. First, between optical zoomed photos, the texture blur & noise balance is the key quality issue, which is determined by the imaging sensor size. The smaller sensor has lower signal-to-noise ratio (SNR). Thus, its rendered texture will be either grainy or muddy depending on whether and how much noise reduction algorithms are applied [37]. The photo from bigger sensor is often clearer in details.

Second, between optically and digitally zoomed images, the blur itself is the biggest difference, which can be modeled by a global point spread function (PSF). Third, between digitally zoomed images, the post-processing algorithm plays a vital role. The ideal result is to add details without introducing annoying artifacts such as ringing, or over-sharpening appearances.

However, current image quality databases mainly simulate the second scenario, i.e., the original image is convolved with a gaussian PSF. The LIVE database [38] collects 29 original images and filters them through a circular-symmetric 2-D gaussian kernel whose standard deviation ranges from 0.42 to 15 pixels. The TID2008 and TID2013 database [39,40] contain 25 reference images and four or five levels of blur distortions. The LIVE MD database [41] improves LIVE or TID by partly simulating the first quality difference scenario: independently and identically distributed gaussian noises are added to the Gaussian blurred image to mimic realistic ISP output.

The BID database [42] is the first attempt to consider realistic blur distortions. Totally, there are 585 images of five classes: unblurred, out-of-focus, simple motion, complex motion and other types. Following BID, Liu et al. [25] investigated the out-of-focus type more deeply. The 150 out-of-focus photos were created by manually focusing a DSLR elsewhere (e.g., at background objects).

Although motion blur and out-of-focus distortion are more realistic than gaussian blur, both rarely happen in the image acquisition process, if not intentionally. For motion blur, the shutter speed is often faster than 1/100 sec in daylight, while in night time, the optical image stabilization (OIS) can help to compensate hand shakes. For phone units without OIS, a safe shutter speed is often enforced by manufacturers (e.g., 1/15 s) to avoid motion blur. As for the out-of-focus problem, first, the depth-of-field of mobile cameras is very thick due to small sensors, that is, the out-of-focus or bokeh phenomenon is not prominent as in the DSLRs. Second, with the advanced dual PDAF and laser autofocus technique, fewer and fewer photos suffer from focusing issues these days. Practically, the low-light environment and moving objects are two probable scenarios where the motion blur or the autofocus system fail, but neither BID [42] nor the out-of-focus database [25] have considered that.

**Figure 2:**
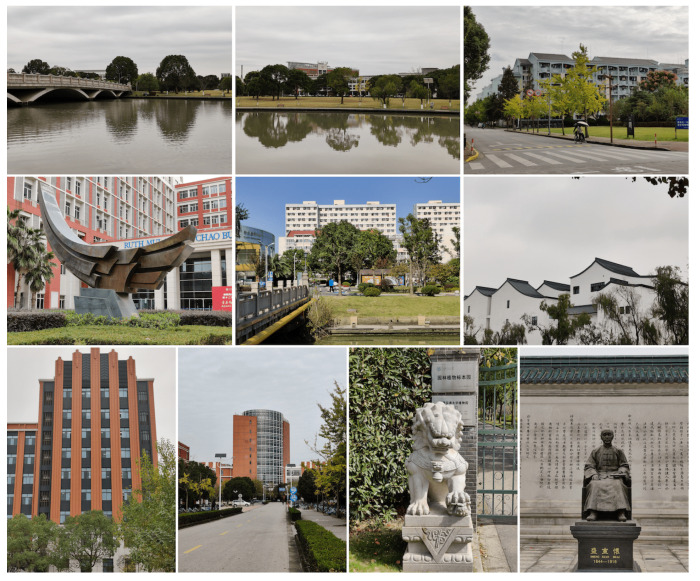
Example zoom photos in our database.

In comparison with motion blur and out-of-focus, blur induced by zooming is much more common and susceptible to smartphones, since no current smartphone cameras possess continuous zooming capability as DSLRs. To build such a zoom photo database, we first select 20 mobile cameras from the market, which include all kinds of brands and span from mid-rangers to high-end models. A large majority of them own at least one zoom lens, while others don’t. Then 45 test scenes are carefully chosen, which include distant sceneries, buildings, characters, portraits as main subjects. During the shooting process, we only zoom in at 2, 3 or 5 times according to the photo composition rule [43]. All grid lines are opened to align image perspectives across different cameras. It is worth noting that no external lenses are mounted on smartphones, and all photos are straight out of the camera without filters and retouches. Finally, each scene is simultaneously captured by all cameras, producing a total number of 900 images. Example images of this Zoom PHoto Database (which we name ZPHD) are shown in Figure 2.

### 2.2. Subjective Quality-Evaluation Study and Analysis

In our pivot subjective study, we found that naïve observers often mistake unnatural textures and over-rate them. Thus, only 10 experts who have experiences in phone camera quality testing are involved. Since we mainly care about the zoomed detail quality, subjects are asked to rate photos by the clarity, ignoring their brightness variations and color differences [44]. According to the recommendations by ITU-R BT.500-12 [45], the test image should be fully shown and the viewing distance be fixed at three times the image height. However, first, our photo size is too large to be displayed in full-screen; second, to differentiate subtle texture differences, more vision acuity (or higher visual angle/spatial frequency) is required [46]. Thus, we allow observers to magnify the test image by themselves for pixel-peeping purposes. It may be questioned that the compared region and magnifying ratio vary across images and subjects. However, except few cases where images from different lenses are stitched together, the clarity of a zoom image does not change dramatically across its patches. And although the magnifying factor is not controlled, the subject tends to compare image details at the same scale. The ambient light is kept low to reduce fatigue.

Moreover, photos from the same scene are grouped together and rated in a session, instead of in completely random order. The subject first views through the whole set (e.g., 20 photos) to form a general idea of the scene content and quality range. Then he/she gives the opinion score based on the single stimulus method [45]. Score of 100 means sharp and clear images while 0 represents heavily pixelated or blurry ones. This is what we improves from our previous work, where only quality rank orders are provided [47]. Table 1 lists major information about the test conditions. Many items have been updated from traditional setups to adapt to our zoom quality applications, and highlighted in boldface.

**Figure 3:**
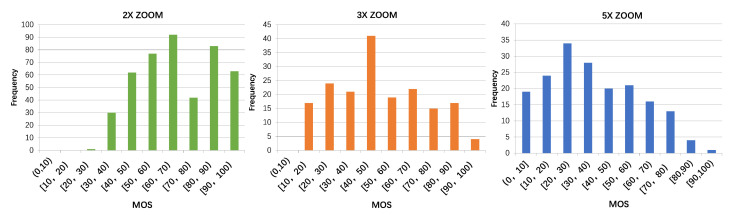
MOS histograms of 2×, 3× and 5× zoom photos (from left to right).

    After obtaining the individual scores, we check the inter-subject consistency using the Spearman correlation coefficient and the Cronbach’s Alpha reliability coefficient [48]. Both results (*r* = 0.943, α=0.957) indicate high correlations and reliability of the subjective scores. Therefore, no second alignment study is performed. Finally, the outlier screening procedure is conducted as in [45]. No scores are found abnormal, so we average all ten scores to obtain the final MOS. The MOS histograms of the 2×, 3×, 5× photos are shown in Figure 3. First, we can observe that, both MOS distributions of 2× and 5× photos are shorter-tailed than 3×’s, i.e., more MOSs are concentrated on either side of the score scale. The reason is that, for most 2× zoom photos, the image quality is still very acceptable (>40) even by the digital zoom of the main camera, not to mention those with 2× optical lenses. While for the 5× zoom photos, except few phone units with 5× periscope, the image quality of the digital zoom by the main camera degrades heavily. Hence, the 5× zoom MOS histogram is more concentrated at the lower end. Second, the overall MOS distribution shifts toward higher value when zoom times becomes larger, this is because, for a single main camera, the 5× zoom photo quality is definitely worse than that of 2× zoom. We also analyze the MOS distribution correlations between difference scenes (of the same zoom times). The r>0.9 indicates that the influence of different scene contents is minor, but the camera with edge-preserving smoothing operation  [49,50] indeed obtains higher MOSs in scenes of characters, architectures than textures. The overall scores are also computed for each camera, which are included in the database.

At last, we summarize characteristics of this zoomed photo database with other representative databases in Table 2. It can be seen that our database contains the largest number of scenes and blur-related images. The image resolution is also the highest.

## 3. Methodology

### 3.1. The Image Sharpness Measurement

As the theoretical foundation, free-energy principle is first introduced in this section. Then the brain interpretation process is approximated using sparse representation. Based on these two models, we deduce the whole formulation of the free-energy and propose a novel sharpness metric, which considers both energy and entropy of the predicted and residual image. Details are given below.

#### 3.1.1. Formulation of Free-Energy Principle

A basic premise of the free-energy-based brain theory [52] is that the cognitive process is governed by an internal generative model. With this internal model, the brain is able to generate the corresponding predictions for its encountered visual scenes, and direct our actions accordingly. For operational amenability, let G denote the brain internal model, and let θ represent the model parameter vector which can be adjusted by G to explain perceived scenes. Given an image *I*, we define its ‘surprise’ or entropy by integrating the joint distribution P(I,θ) reciprocal over the space of the parameter vector θ:

(1) $$\begin{array}{c} -\log P(I)=-\log\int P(I,\theta )d\theta \end{array}$$

Since the P(I,θ) is too complicated to write analytically, we introduce an auxiliary term Q(θ|I) into both the denominator and numerator of the right part in Equation (1) and have:

(2) $$\begin{array}{c} -\log P\left(I\right)=-\log\int Q\left(\theta |I\right)\frac{P(I,\theta )}{Q(\theta |I)}d\theta \end{array}$$

Here Q(θ|I) is the posterior distribution of the model parameters given image *I*, which can be thought of as an approximate posterior to the true posterior of the model parameters P(θ|I) calculated by the brain. When perceiving the input image *I*, the brain intends to minimize the discrepancy between the approximate posterior Q(θ|I) and the true posterior P(θ|I). In fact, this approximation technique has been used in ensemble learning and variational Bayesian estimation framework. Please see [53] for more details.

By using Jensen’s inequality, we can move the logarithm operation inside the integral, thus Equation (2) can be translated into:

(3) $$\begin{array}{c} -\log P\left(I\right)\leq -\int Q\left(\theta |I\right)\log\frac{P(I,\theta )}{Q(\theta |I)}d\theta \end{array}$$

According to statistical physics and thermodynamics [54], the right side of Equation (3) is defined as “free energy” as follows:

(4) $$\begin{array}{c} F\left(\theta \right)=-\int Q\left(\theta |I\right)\log\frac{P(I,\theta )}{Q(\theta |I)}d\theta \end{array}$$

Obviously, F(θ) defines an upper bound of `surprise’ for image *I*. In practice, the integration over the joint distribution P(I,θ) can be intractably complex. By decomposing P(I,θ)=P(θ|I)P(I), we obtain:

(5) $$\begin{array}{cc} F(\theta ) & =\int Q\left(\theta |I\right)\log\frac{Q(\theta |I)}{P(\theta |I)P(I)}d\theta \\ \; & =-\log P\left(I\right)+\int Q\left(\theta |I\right)\log\frac{Q(\theta |I)}{P(\theta |I)}d\theta \\ \; & =-\log P(I)+\mathrm{KL}(Q(\theta |I)∥P(\theta |I)) \end{array}$$

where KL(·) refers to the Kullback-Leibler divergence between the approximate posterior and the true posterior distributions and it’s nonnegative. It is clearly seen that the free energy F(θ) is greater than or equal to the image `surprise’, −logP(I). In visual perception, the brain tries to minimize KL(Q(θ|I)∥P(θ|I)) of the divergence between the approximate posterior and its true posterior distributions when perceiving image *I*.

Alternatively, noticing that P(I,θ)=P(I|θ)P(θ), (4) can be rewritten as:

(6) $$\begin{array}{cc} F(\theta ) & =\int Q\left(\theta |I\right)\log\frac{Q(\theta |I)}{P(I|\theta )P(\theta )}d\theta \\ \; & =\int Q\left(\theta |I\right)\log\frac{Q(\theta |I)}{P(\theta )}d\theta -\int Q\left(\theta |I\right)\log P\left(I|\theta \right)d\theta \\ \; & =\mathrm{KL}\left(Q\left(\theta |I\right)∥P\left(\theta \right)\right)+E_{Q}\left[\log\frac{1}{P(I|\theta )}\right] \end{array}$$

In the next two subsections, we will explain how to calculate the two parts in the right hand side of Equation (5) or (6), and leverage both of them for the sharpness estimation.

#### 3.1.2. Approximation of the Brain Generative Model

For the application of free-energy theory into the image quality assessment, the concrete form of G needs to be specified first. In [55], the receptive fields of simple cells in mammalian primary visual cortex are characterized as spatially localized, oriented and bandpass. Sparse representation mimic the above biological process by assuming that the image or its patch can be modeled by a linear combination of few atoms from a predefined or trained dictionary [56]. Such atoms have been evidenced to resemble neural response characteristics well, and the superiority of sparse representation for approximating the internal model has been verified in [57]. Therefore, we utilize the sparse coding as the deputy of model G.

Mathematically, given an image *I*, a patch $x_{k}\in R^{B}$ of size $\sqrt{B}\times \sqrt{B}$ is extracted from *I* by:

(7) $$\begin{array}{c} x_{k}=R_{k}\left(I\right) \end{array}$$

where $R_{k}\left(\cdot \right)$ simply copies the pixel values from image *I* at location *k* into $x_{k}$, k=1,2,3...N. *N* is the total number of image patches.

For the specific extracted patch $x_{k}$, its sparse representation over a dictionary $D\in R^{B\times d}\;\left(d>B\right)$ refers to finding a sparse vector $\alpha_{k}\in R^{d}$ (i.e., most of the elements in $\alpha_{k}$ are zero or close to zero) to satisfy:

(8) $$\begin{array}{c} x_{k}=D\alpha_{k} \end{array}$$

If small approximation errors are permitted, the exactly equal relation of (8) can be relaxed as:

(9) $$\begin{array}{c} ∥x_{k}-D\alpha_{k}{∥}_{p}\leq \xi \end{array}$$

where ${∥\cdot ∥}_{p}$ refers to the $l^{p}$-norm. ξ is a positive number. Because our objective is the sparsity of coefficients, the constrained problem can be formulated as:

(10) $$\begin{array}{ccc} \alpha_{k}^{*}=\underset{\alpha_{k}}{\mathrm{argmin}}∥\alpha_{k}{∥}_{p} & s.t. & ∥x_{k}-D\alpha_{k}{∥}_{p}\leq \xi \end{array}$$

**Figure 4:**
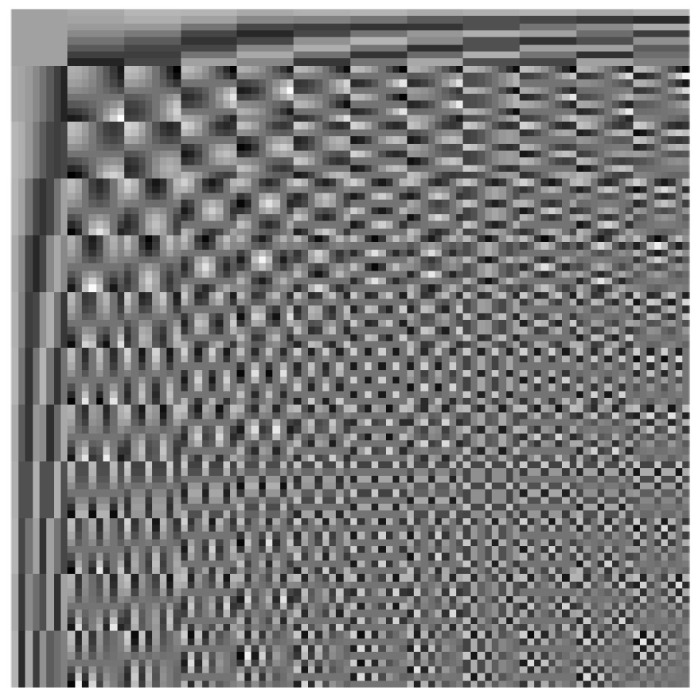
The 144 atoms of 2D-DCT over-complete dictionary.

Alternatively, the dual problem of (10) could be considered:

(11) $$\begin{array}{ccc} \alpha_{k}^{*}=\underset{\alpha_{k}}{\mathrm{argmin}}{∥x_{k}-D\alpha_{k}∥}_{p} & s.t. & ∥\alpha_{k}{∥}_{p}\leq \delta \end{array}$$

where δ is the threshold of sparsity. Both (10) and (11) can be transformed into an unconstrained optimization problem:

(12) $$\begin{array}{c} \alpha_{k}^{*}=\underset{\alpha_{k}}{\mathrm{argmin}}\frac{1}{2}∥x_{k}-D\alpha_{k}{∥}_{2}+\lambda {∥\alpha_{k}∥}_{p} \end{array}$$

where the first term is the reconstruction fidelity constraint and the second term is to punish the sparsity of the representation coefficient vector. λ is a positive constant to weigh the importance of these two terms. When p=0, the sparsity of $\alpha_{k}$ is controlled by the $l^{0}$-norm. An alternative way is to replace the $l^{0}$-norm with $l^{1}$-norm, which is convex and can be solved by iterative shrinkage/thresholding algorithm [58]. We employ off-the-shelf 2-D DCT bases as our predefined dictionary D, which is illustrated in Figure 4. More implementation details can be found in the Section 4.

After obtaining the sparse coefficient vector $\alpha_{k}^{*}$ for the image patch $x_{k}$, we substitute $x_{k}$ with $D\alpha_{k}^{*}$, then copies it back into the original position by:

(13) $$\begin{array}{c} I^{\prime }=\sum_{k=1}^{n}R_{k}^{T}\left(D\alpha_{k}^{*}\right)./\sum_{k=1}^{n}R_{k}^{T}\left(1_{B_{s}}\right) \end{array}$$

where $R_{k}^{T}$ is the reverse operator of $R_{k}$; and $I^{\prime }$ refers to the sparse representation for the image *I*, or the brain prediction result in the free-energy theory.

#### 3.1.3. The Sharpness Index

In [36], the mathematical expression of free-energy has been derived for AR model and applied for RR-IQA tasks. In this paper, the sparse representation coefficients α become the model parameter θ, and the first term of (6), KL(Q(α|I)∥P(α)) measures the distance between the recognition density and the true prior density of model parameters. However, although the support (i.g. non-zero position) of α and the distribution of P(α) have been discussed in structured compressed sensing [59,60], their exact forms are not determined yet. To simplify computation, we choose Gaussian distribution for the prior density P(α). This Gaussian prior rightly corresponds to $l^{2}$-norm relaxation of the $l^{0}$-norm in (12), and can be transformed into an inverse Bayesian inference problem [56]. To model the recognition density Q(α|I), i.e., the posterior distribution of 2D-DCT sparse coefficients, we use Gaussian function as well.

Specifically, let P(α)=N($\alpha_{p}$; $\mu_{p}$, $\Sigma_{p}$), and Q(α|I)=N($\alpha_{q}$; $\mu_{q}$, $\Sigma_{q}$), the KL-divergence becomes:

(14) $$\begin{array}{c} \mathrm{KL}\left(Q\left(\alpha |I\right)∥P\left(\alpha \right)\right)=\frac{1}{2}(\log\frac{|\Sigma_{p}|}{|\Sigma_{q}|}-d+Tr\left(\Sigma_{p}^{-1}\Sigma_{q}\right)\\ +{\left(\mu_{q}-\mu_{p}\right)}^{T}\Sigma_{p}^{-1}\left(\mu_{q}-\mu_{p}\right)) \end{array}$$

where *d* is the dimension of variable α. If we further assume sparse coefficient vector $\alpha_{p}=\left[\alpha_{1},...,\alpha_{d}\right]$ is uncorrelated, (14) can be simplified as:

(15) $$\begin{array}{c} \mathrm{KL}\left(Q\left(\alpha |I\right)∥P\left(\alpha \right)\right)=\frac{1}{2}{\left(\mu_{q}-\mu_{p}\right)}^{T}diag\left(\lambda_{1}^{-1},...,\lambda_{d}^{-1}\right)\left(\mu_{q}-\mu_{p}\right)+const \end{array}$$

where $\left(\lambda_{1},...,\lambda_{d}\right)$ are variances of $\alpha_{p}$, $const=\frac{1}{2}(\log\frac{|\Sigma_{p}|}{|\Sigma_{q}|}-d+Tr\left(\Sigma_{p}^{-1}\Sigma_{q}\right)$. This equation manifests that, the KL-divergence can be calculated through the quadratic sum of sparse coefficients, divided by the variance components of α.

The second term of (6), $E_{Q}\left[\log\frac{1}{P(I|\alpha )}\right]$, measures the average likelihood of the P(I|α) under Q(α|I). If we approximate Q(α|I) with P(I|α), then

(16) $$\begin{array}{c} E_{Q}\left[\log\frac{1}{P(I|\alpha )}\right]=\int Q\left(\alpha |I\right)\log\frac{1}{P(I|\alpha )}d\alpha \approx \int P\left(I|\alpha \right)\log\frac{1}{P(I|\alpha )}d\alpha \\ =E(\log\frac{1}{P(I|\alpha )}) \end{array}$$

By combining (15) and (16), the free-energy quantity can be written as:

(17) $$\begin{array}{cc} F(\alpha ) & =\mathrm{KL}\left(Q\left(\alpha |I\right)∥P\left(\alpha \right)\right)+E_{Q}\left[\log\frac{1}{P(I|\alpha )}\right]\\ \; & \approx \frac{1}{2}{\left(\mu_{q}-\mu_{p}\right)}^{T}diag\left(\lambda_{1}^{-1},...,\lambda_{d}^{-1}\right)\left(\mu_{q}-\mu_{p}\right)+E\left(\log\frac{1}{P(I|\alpha )}\right) \end{array}$$

which means free-energy equals the model approximation error plus image `surprise’. This factorization can also be seen through (5), where the first term −logP(I) defines the log-evidence of the image, which is just the negative of `surprise’; and the second term KL(Q(θ|I)∥P(θ|I)) measures the KL-distance between approximate model density and true posterior density.

In implementation, we set $\mu_{p}=0$ (smooth prior), $\mu_{q}=\alpha_{k}^{*}$, and further compress the variance vector into a single scalar λ, which is computed through the *k*-th image patch variances $\sigma_{k}^{2}$. The $\sigma_{k}^{2}$ also serves as the compensation of contrast masking effect induced by image contents and has been proved effective in previous works [13,23]. The entropy is calculated in the residual domain. To balance different scales of the model error term and the entropy, we also introduce a weighing coefficient $k_{1}$. Thus, (17) is simplified as:

(18) $$\begin{array}{c} F\left(\alpha \right)\approx \frac{{\left(\alpha_{k}^{*}\right)}^{T}\alpha_{k}^{*}}{\sigma_{k}^{2}}+k_{1}\cdot E\left(\log\frac{1}{P(\left(I-I^{\prime }\right)|\alpha )}\right) \end{array}$$

where $I^{\prime }$ is the reconstructed image by sparse coding.

Since human visual system is more sensitive to sharper regions, it is effective to only pool them together [15]. To capture image structures and details efficiently, the gradient domain is used. Let ∇I denotes the gradient magnitude of image *I* by Sobel edge detector, S⊙ is the binary operator which assigns 1s for the top l% sharpest patches (0s otherwise), and $N_{s}=\left\lceil l\%\times N\right\rceil$ denotes the number of these sharpest blocks, where *N* is the total number of image patches. We define the sharpness index as:

(19) $$\begin{array}{c} SS=\sum_{k=1}^{N_{s}}\frac{{\left(\alpha_{k}^{*}\right)}^{T}\alpha_{k}^{*}}{\sigma_{k}^{2}}+k_{1}\cdot E\left(\log\frac{1}{P(S⊙(|\nabla I-\nabla I^{\prime }\left|)|\alpha \right)}\right) \end{array}$$

Looking at (18, 19), our work is the first to give the complete formulation of free-energy under the sparse representation model and leverage its full power for sharpness assessment. In contrast, Wu et al. [61] interpreted the predicted and residual image as ordered and disordered portion. The latter is regarded useless for high-level inference and mainly responsible for distortions such as noise, compression artifacts, etc. Gu et al. [22] decomposed the blurred image through the autoregressive model. The AR-predicted result is a re-blurred version of the test image and used for the sharpness estimation afterwards, but the residual part is not considered. Although the residual part is used in [62], the reconstructed image is ignored. In our paper, the reconstructed part represents the prominent structure/edges of the image. And decent amounts of fine-grained details are left in the residual image, which are crucial in the differentiation of small quality gaps between zoom photos.

**Figure 5:**
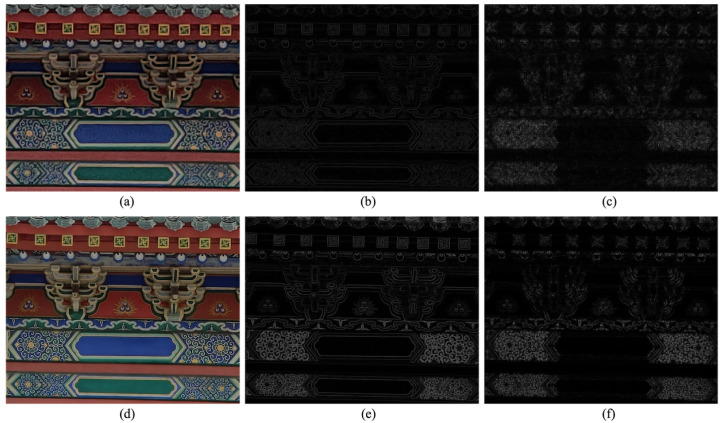
Demonstration of the effectiveness of both energy and entropy for sharpness. The first column shows two image crops from our 3× zoom database by digitally and optically zooming, while the second and third columns contain their predicted gradient and residual images, respectively (we have extended the dynamic range of the residual image for a clearer view, which has no influence on the computed entropy). (**a**) MOS = 43.6. (**b**) KL= 4.2008. (**c**) Entropy = 2.3151. (**d**) MOS = 87.4. (**e**) KL = 6.0919. (**f**) Entropy = 3.2535.

Figure 5a,d compare two 3× zoom photo crops. The MOS of Figure 5a is higher than Figure 5d because of hardware advantage. In their sparse reconstructed gradient images, the edge intensity of eaves and decorative patterns in Figure 5e are much stronger than Figure 5b, which is reflected in the KL-divergence (${\mathrm{KL}}_{b}$ = 4.2008, ${\mathrm{KL}}_{e}$ = 6.0919). Meanwhile, the residuals in Figure 5f are also more obvious and uneven than Figure 5c (see the decorative patterns), leading to a higher entropy as well. Therefore, both the energy and entropy of the predicted and residual portions can represent the sharpness changes.

Moreover, we can observe that Figure 5b,e primarily capture the intensity of edges (i.e., acutance), while Figure 5c,f depict the subtle and fine-grained textures (i.e., resolution). Acutance and resolution are two complementary factors in the perception of sharpness [17], both of which form an integral part of our sharpness model.

### 3.2. The Image Naturalness Measurement

Although the sharpness metric can distinguish quality differences between optical and digital lenses, it easily over-estimates the over-sharpening effect and spurious artifacts present in the zoom photos due to the the post-processing algorithms. The image naturalness, by its name, can measure such loss of naturalness and degradation of the perceptual quality. In literature, different forms of the natural scene statistics have been used: the power law of the spectrum energy is measured in [9,17]; the non-gaussian distribution of the gradient component, DCT or Fourier coefficients are used in [63,64] for noise estimation and quality assessment; the Rayleigh or Weibull distribution of the variance or gradient magnitude are leveraged in [33,65]. Here, we utilize the distribution of mean subtracted contrast normalized (MSCN) coefficients as in [31,32].

Specifically, let *x* and *y* denote the pixel coordinates, and I(x,y), μ(x,y), σ(x,y) refer to the original images, mean and standard deviation of the local image patch centered at (x,y), respectively, then the MSCN coefficient $\hat{I}\left(x,y\right)$ at (x,y) is defined as:

(20) $$\begin{array}{c} \hat{I}\left(x,y\right)=\frac{I(x,y)-\mu (x,y)}{\sigma (x,y)+1} \end{array}$$

According to [66], the image structure transitions are reduced due to this local non-linear divisive normalization, and for pristine natural images, the highly leptokurtic and long-tailed characteristics are transformed into a unit normal Gaussian distribution. However, for over-sharpened photos, the abrupt details would change the behavior of both peaks and tails of the empirical coefficient distribution, which can be well modeled by a generalized Gaussian distribution (GGD):

(21) $$\begin{array}{c} g\left(x;\alpha ,\beta \right)=\frac{\alpha }{2\beta \Gamma (1/\alpha )}\exp\left(-{\left(\frac{\left|x\right|}{\beta }\right)}^{\alpha }\right) \end{array}$$

where Γ(·) refers to the gamma function, which is defined as:

(22) $$\begin{array}{c} \Gamma \left(x\right)=\int_{0}^{\infty }\phi^{x-1}e^{-\phi }d\phi ,\;x>0 \end{array}$$

where α and β are the GGD parameters, which can be estimated by the moment matching-based method [67].

**Figure 6:**
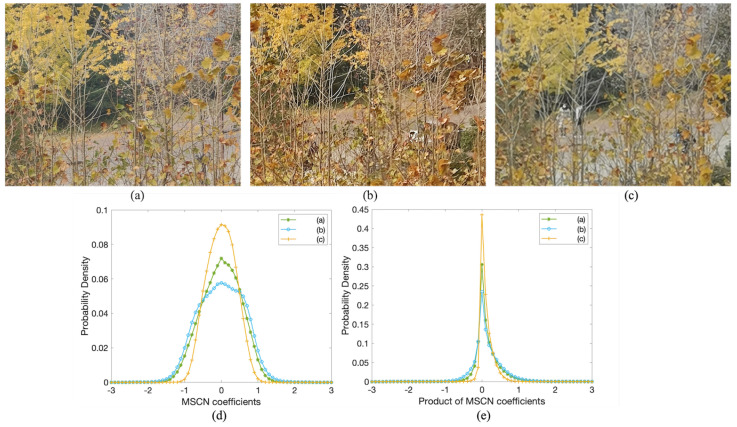
(**a**–**c**) are three crops of high-quality, over-sharpening, zoom blur photos, respectively; (**d**,**e**) show histograms of their MSCN and product of horizontal MSCN coefficients.

In order to better visualize the effects of over-sharpening and zoom blur, Figure 6a shows a natural-looking photo crop from our 5× zoom database, while Figure 6b,c are two other photos from the same scene that suffer from over-sharpening problems and slight blur, respectively. Figure 6d plots the corresponding empirical distributions of Figure 6a–c. It is observed that the MSCN coefficients of Figure 6a follow a uniform Gaussian distribution, while Figure 6b reduces the weight of the tail of the histogram and Figure 6c appears to exhibit a more Laplacian appearance. Instead of calculating sample statistics such as the variance, skewness or kurtosis [10,68], we directly use α and β to encompass a wider range of distortion changes.

Aside from the MSCN coefficients, the products of the adjacent MSCN coefficient pairs are also powerful to characterize the image quality. Figure 6e shows the empirical distribution of the horizontal product MSCN coefficients of Figure 6a–c. As we can see, the histogram of Figure 6c is more peaked and leptokurtic than Figure 6a, while the distribution shape of Figure 6b looks more flat-topped. In this paper, we calculate the products of the adjacent MSCN coefficients along four directions, i.e., horizontal, vertical, main-diagonal and second-diagonal as in the  [31]. Each of these products can be modeled with the zero-mode asymmetric GGD (AGGD):

(23) $$g\left(x;\gamma ,\beta_{l},\beta_{r}\right)=\left\{\begin{array}{c} \frac{\gamma }{\left(\beta_{l}+\beta_{r}\right)\Gamma \left(\frac{1}{\gamma }\right)}\exp\left(-{\left(\frac{-x}{\beta_{l}}\right)}^{\gamma }\right)\;\forall x\leq 0\\ \frac{\gamma }{\left(\beta_{l}+\beta_{r}\right)\Gamma \left(\frac{1}{\gamma }\right)}\exp\left(-{\left(\frac{x}{\beta_{r}}\right)}^{\gamma }\right)\;\forall x>0 \end{array}\right.$$

Unlike MSCN, the mean of the product MSCN distributions also differs for Figure 6a–c, indicating the changes of zoom quality. Thus, we compute the mean as:

(24) $$\begin{array}{c} \eta =\left(\beta_{r}-\beta_{l}\right)\frac{\Gamma (\frac{2}{\gamma })}{\Gamma (\frac{1}{\gamma })} \end{array}$$

The informative model parameters $\left(\gamma ,\beta_{l},\beta_{r},\eta \right)$ of the AGGD are estimated and introduced into our quality-aware NSS features. As research in quality assessment has demonstrated that incorporating multi-scale information correlates better with human perception [69,70], we extract the above features in two scales (low-pass filtered and downsampled by 2).

Instead of calculating feature vectors on the whole image, we partition the photo into non-overlapping patches and perform feature extraction on each of them, leading to a 36-dimensional vector for each patch. Then we stack all the feature vectors together and fit them with a multivariate Gaussian (MVG) density as:

(25) $$\begin{array}{c} f\left(x\right)=\frac{1}{{\left(2\pi \right)}^{k/2}{\left|\Sigma_{x}\right|}^{1/2}}\exp\left\{-\frac{1}{2}{\left(x-\mu_{x}\right)}^{T}\Sigma_{x}^{-1}\left(x-\mu_{x}\right)\right\} \end{array}$$

where x refers to the quality feature vector and *k* refers to the dimension of x.

To learn a model that serves as the pristine anchor for the NSS features, we select one hundred pristine images from the Berkeley image segmentation database [71], then model their patch-based feature vectors using MVG as well:

(26) $$\begin{array}{c} f\left(y\right)=\frac{1}{{\left(2\pi \right)}^{k/2}{\left|\Sigma_{y}\right|}^{1/2}}\exp\left\{-\frac{1}{2}{\left(y-\mu_{y}\right)}^{T}\Sigma_{y}^{-1}\left(y-\mu_{y}\right)\right\} \end{array}$$

A common measure between two distribution distances is the KL-divergence. However, the KLD is asymmetrical. In this paper, we use the square root of the symmetric Jenson-Shannon (JS) divergence [72] to define our unnaturalness score:

(27) $$\begin{array}{c} JS\left(f\left(x\right),f\left(y\right)\right)=\mathrm{KL}\left(f\left(x\right)∥\frac{f(x)+f(y)}{2}\right)+\mathrm{KL}\left(f\left(y\right)∥\frac{f(x)+f(y)}{2}\right) \end{array}$$

(28) $$\begin{array}{c} NS=\sqrt{{\left(\mu_{x}-\mu_{y}\right)}^{T}{\left(\frac{\Sigma_{x}+\Sigma_{y}}{2}\right)}^{-1}\left(\mu_{x}-\mu_{y}\right)} \end{array}$$

where NS measures the distance between the tested zoom photos and pristine images, thereby representing the inverse of image naturalness. The smaller NS is, the more natural a zoom photo appears.

### 3.3. The Final Zoom Quality Metric

**Figure 7:**
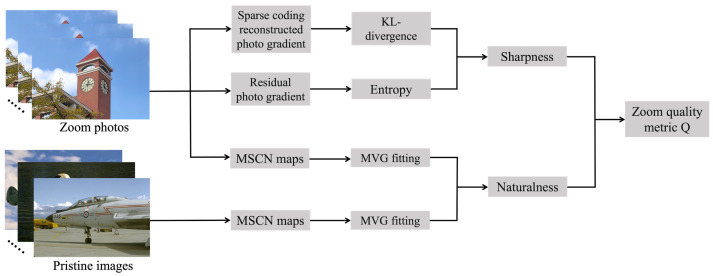
Flowchart of the proposed zoom quality metric.

After calculating the sharpness and image naturalness, we attempt to merge them into a single zoom quality index. We found that a simple linear combination is enough to yield good results. Other weighting strategies such as geometric weighting, Boltzmann machine and SVM regression can achieve similar results, but their interpretability is not as good as linear combination and may suffer from over-fitting problems. Thus, the final zoom quality metric *Q* is defined as:

(29) $$\begin{array}{c} Q=SS+w\cdot NS \end{array}$$

where *w* is a negative constant that determines the relative importance of SS and NS. We will discuss it more deeply in the Section 4. For intuitive understanding of the proposed zoom quality metric, we show its flowchart in Figure 7.

## 4. Experiments

### 4.1. Implementation Details

In implementation, the zoom photo is divided into 8 x 8 non-overlapping patches. Then we reshape the square patch into a 64 × 1 column, which constitutes $x_{k}$ in Equation (7). To construct the 2D-DCT dictionary, we first create a 1D-DCT matrix $A_{1D}$ of size 8 × 12, where the *k*-th atom (*k* = 1,2,…,12) is given by $a_{k}=\cos\left(\left(i-1\right)\left(k-1\right)\pi /12\right)$, *i* = 1,2,…,8. Then all the atoms except the first constant one are processed by removing their mean. The final 64 x 144 over-complete dictionary D is obtained by a Kronecker-product $D=A_{1D}⊗A_{1D}$. The non-convex $l^{0}$-minimization problem (10, 11) is solved using the orthogonal matching pursuit (OMP) algorithm [73]. We set the sparsity degree at 6 experimentally. The l=60%, $k_{1}=0.5$ and w=−0.7 are optimized to achieve the top result on the zoom photo database.

To easily follow the process of this zoom quality metric, we show its pseudo code in the Algorithm 1 below.    
**Algorithm 1:** Pseudo-code of the proposed zoom quality metric**Input**: Zoom photo *I*, over-completer DCT dictionary **D**, mean and variances of pristine MVG parameters $\mu_{y}$, $\Sigma_{y}$, weighting parameters $k_{1}$, *w* and *l*.1**Initialization:**2Compute the photo gradient ∇I using Sobel operator;3Partition the photo *I* into non-overlapping 96 x 96 patches $x_{k}$
4**The measurement of sharpness:**5**foreach **k=1,2,…,N**do**6   Solve the sparse coding coefficients $\alpha_{k}^{*}$ using the OMP algorithm [73];7  Calculate the patch variance $\sigma_{k}^{2}$;8  Sort and select the top l% patches according to the variance $\sigma_{k}^{2}$;9**end**(10Compute mean KL-divergence or energy: $\sum_{k=1}^{N_{s}}\frac{{\left(\alpha_{k}^{*}\right)}^{T}\alpha_{k}^{*}}{\sigma_{k}^{2}}$
11Compute the residual gradient image: $\nabla I-\nabla I^{\prime }$
12Compute entropy of the residual: $E(\log\frac{1}{P(S⊙(|\nabla I-\nabla I^{\prime }\left|)|\alpha \right)})$ 
13**The sharpness index:** $SS=\sum_{k=1}^{N_{s}}\frac{{\left(\alpha_{k}^{*}\right)}^{T}\alpha_{k}^{*}}{\sigma_{k}^{2}}+k_{1}\cdot E\left(\log\frac{1}{P(S⊙(|\nabla I-\nabla I^{\prime }\left|)|\alpha \right)}\right)$ 
14**The measurement of naturalness:**15**foreach **k=1,2,…,N **do**((16 Compute the MSCN map of *I* using (20)17 Compute the GGD and AGGD parameters of each patch using (21), (23), (24)18**end**(19Estimate the $\mu_{x}$ and $\Sigma_{x}$ through (25)20**The naturalness index:**  $NS=\sqrt{{\left(\mu_{x}-\mu_{y}\right)}^{T}{\left(\frac{\Sigma_{x}+\Sigma_{y}}{2}\right)}^{-1}\left(\mu_{x}-\mu_{y}\right)}$ **Output:** Zoom quality metric Q=SS+w·NS

### 4.2. Illustrative Results

**Figure 8:**
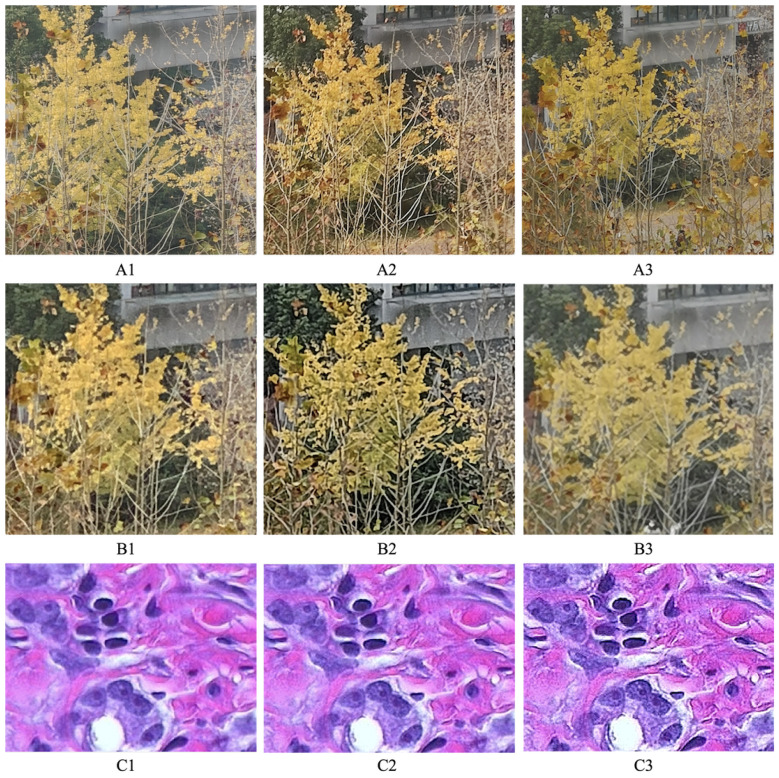
Three scenarios where both sharpness and naturalness fail to predict the image quality, but our zoom quality metric can. The detailed explanation is in the main text.

**Figure 9:**
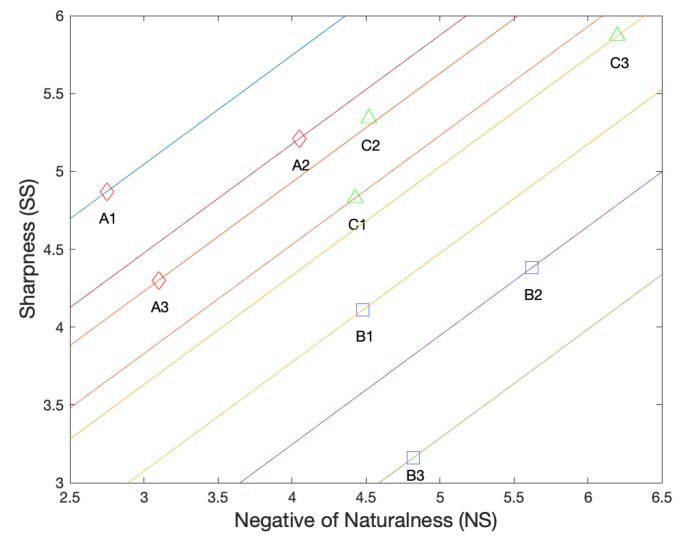
Two dimensional plots of naturalness versus sharpness for example images in Figure 8 (A1–C3). Each straight line represents the same level of *Q* and the top-left one indicates a higher *Q*. The line passing through C2 almost overlaps with that of A3, thus is omitted for a better view.

Before quantitative results, let us examine three scenarios where our zoom quality metric succeeds while single SS or NS fails to predict the image quality in Figure 8:

1 In its top row, the quality of A1 > A2 > A3. Specifically, A1 is a 5× zoom photo taken with an optical camera, while A2 comes from a smaller sensor and looks more grainy. A3 is interpolated from a 3× zoom camera, which suffers from zoom blur. From Figure 9, we can observe that the sharpness score (i.e., SS) wrongly judges A2 > A1 > A3, and the naturalness score (i.e., −NS) mistakes A1 > A3 > A2. This fact reveals drawbacks of the SS and SS: SS over-estimates the sharpening effect (A2 > A1), while NS over-emphasizes the image naturalness or smoothness (A3 > A2). In contrast, our zoom quality metric, indicated by the level sets of straight lines in Figure 9, successfully gives the order of A1 > A2 > A3;
2 Similarly, in the second row of Figure 8, all B1, B2 and B3 are 5× zoom photos interpolated from three 2× optical lenses but with different ISPs and post-processing algorithms. The quality order is B1 > B2 > B3. However, the SS of B2 is larger than B1 because of annoying artifacts. Although the NS predicts the quality of B1 > B2 without error, it over-estimates the zoom blur (softness) present in B3, thus wrongly judging B3 > B2. By combining the SS with NS, the correct order of B1 > B2 > B3 can be achieved with our metric Q;
3 In the bottom row, C1 is the original “*hestain*” image, and C2 and C3 are created using the Matlab *imsharpen function* with different amounts of unmask sharpening. The quality order is C2 > C1 > C3, as it is well-known that moderate amounts of sharpening can improve an image’s perceptual quality, while excessive sharpening would lead to a more unnatural appearance, thereby degrading the naturalness. However, from Figure 9, we can see the SS scores increase monotonically with the sharpening amounts, that is, C3 > C2 > C1, while the NS penalizes the C2 too much, leading to C1 > C2 > C3. In contrast, our metric *Q* can evaluate them more appropriately (C2 > C1 > C3). This fact implies our zoom quality metric can be used to control the parameter of sharpening algorithms.

### 4.3. Performance Comparison

In this subsection, we compare the proposed quality metric with seventeen state-of-the-art NR-IQA algorithms on the zoom photo database, which can be classified into three categories: sharpness-specific, unsupervised and supervised general-purpose ones. The sharpness metrics include JNB [13], CPBD [15], S3 [17], FISH [18], LPC [19], SPARISH [23] and S3-III [27]. The unsupervised or opinion-free algorithms are NIQE [32], SNP-NIQE [33], IL-NIQE [65], NPQI [74], LPSI [75] and QAC [76]. Belonging to the supervised models are BIQI [77], BRISQUE [31], BLIINDS-II [64] and M3 [78]. Except S3-III [27], all source codes of these algorithms are obtained from original authors or their public websites. We implement the S3-III algorithm [27] by ourselves. The SROCC, KROCC and PLCC are calculated using the protocol suggested by VQEG [79].

Table 3 lists the performance on the our zoom photo database. The best performed method is marked in boldface. It can be seen that the general-purpose NR methods assess the quality of the zoom blurred images moderately due to their general QA ability for distorted images. Compared with the general-purpose methods, the sharpness specific methods achieve better prediction results. This can be verified by the observation that most of the SROCC values of the sharpness metrics are higher than 0.75. Moreover, S3-III [27] doesn’t improve the S3 [17] by a large margin in our database. Last but not least, our proposed zoom quality metric earns superior prediction performance to all of the competing methods and outperforms them remarkably.

### 4.4. The Discussion of w: The Tradeoff between Sharpness and Naturalness

**Figure 10:**
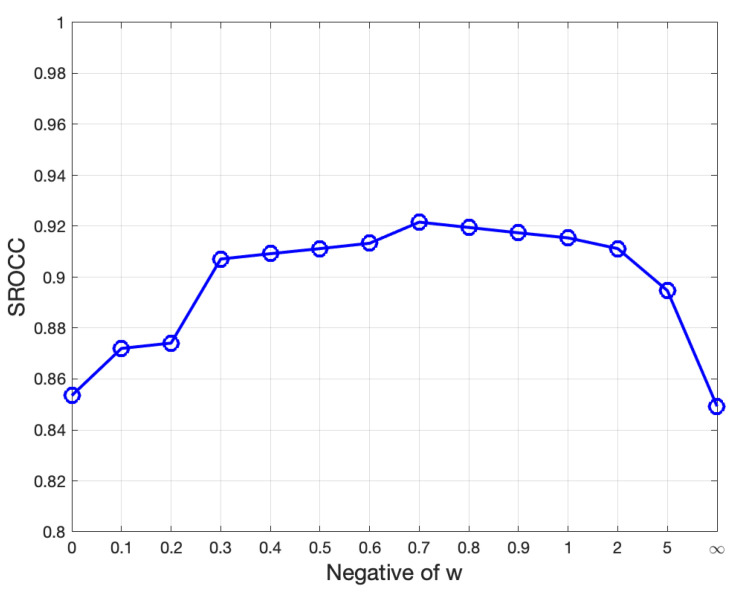
Plot of SROCC trends with different *w*.

The special case of w=0 corresponds to the sharpness measure, while −w=∞ refers to the naturalness measure. Figure 10 plots the SROCC value versus different *w*. It can be seen that, the SROCC increases steeply when −w is in (0–0.3), then reaches a plateau in (0.4–1) and eventually drops down as −w increases to *∞*. This is because a small −w (−w<0.3) only emphasizes the importance of sharpness, ignoring the photo smoothness or naturalness. This is also the drawback of existing sharpness indices, which are not close-looped. At the other end, a very large −w (−w>1) gives more weight to the image softness, which contradicts the common sense that an appropriate sharpening operation can improve the perceptual quality. By choosing an appropriate −w, we can obtain a trade-off between sharpness and naturalness. In this paper, we choose −w=0.7, as it achieves the best result in Figure 10. However, it is worth noting that −w∈(0.4,1) are all reasonable choices, which depend on personal preference. People who tend to prefer a smoother photo may choose a larger w, and vice versa. There have been phone models such as Galaxy S23 series that offer this softness adjustment option.

### 4.5. Limitations of the Current Work

Despite the best result achieved by the proposed metric in the zoom quality database, there exist several limitations: (1) our metric doesn’t consider the influence of color differences and exposure variations. Although detail rendering is perhaps the most determining factor in the zoom photo quality, taking into account other quality aspects is also necessary, since the HDR capability and color rendering in zoom lenses are always not consistent and good as the main camera [47]; (2) the constant *w* could be generalized to a function w(c,q). As we mentioned in Section 2.2, image contents of characters could bear more sharpening amounts than textures, animal fur and people skin. And compared to high-quality photos, images suffering heavy zoom blur could benefit from more sharpening, too. In these two cases, the *w* could be lowered; (3) besides using w(c,q), another way to improve the metric performance is to utilize machine-learning [80], which we will look into in the next future; (4) There has been a trend of using AI-restoration technique, especially for the long-range zoom photos. These AI-generated textures may improve perceptual quality for characters, but the fake, wrinkle-like details would make photo dirty and weird. Currently, our algorithm couldn’t handle the quality degradation of AI-generated textures very effectively.

## 5. Conclusions

Zoom photos differ from gaussian blurred images in their over-sharpening appearances and harmful artifacts. To assess them rightly, we first build a zoom photo database which consists of 20 mobile units and 45 texture-complex scenes. Then we propose a novel zoom quality metric, considering both sharpness and naturalness. To evaluate the sharpness, we are the first to give the whole formulation of free-energy theory under sparse coding, and leverage both the energy and entropy of the predicted and residual images. To measure the naturalness, we extract a set of MSCN coefficients, and then compare it with that of pristine images under the multi-variant Gaussian model. In the experiments, drawbacks of the single sharpness or naturalness are revealed, and the effectiveness of their summation is illustrated by three scenarios. We also discuss different choices of the linear combination coefficient. Finally, the SROCC, KROCC, and PLCC in the zoom photo database demonstrate the superiority of our metric over traditional sharpness and general-purpose methods.

## Figures and Tables

**Table 1:** Configurations of subjective experiments.

Configuration	Values
Evaluation method	**single-stimulus within a scene**
Evaluation scales	continuous quality scales from 0 to 100
Evaluation standard	**image clarity instead of color or overall quality**
Image number	20 mobile cameras × 45 scenes
Image encoder	**JPEG and HEIF**
Image resolution	**mostly 4000 × 3000**
Subjects	**ten experienced experts**
Viewing angle	**can be adjusted by the subject**
Room illuminance	low

**Table 2:** Summary of typical IQA databases having blur-related distortions.

Databases	No. of Ref. Scenes	No. of Dist. Images	No. of Dist. Types	No. of Blurred Levels/Images	Creation Process	Resolution	Subjects No.
LIVE [38]	29	779	5	4/174	Gaussian blur	568 × 712	20–29
TID2018 [39]	25	1700	17	4/100	Gaussian blur	512 × 384	838
TID2013 [40]	25	3000	24	5/125	Gaussian blur	512 × 384	971
CSIQ [51]	30	866	6	5/150	Gaussian blur	512 × 512	25
LIVE MD [41]	15	450	2	15/225	Gaussian blur+wn	1280 × 720	18
BID [42]	N.A.	585	5	N.A./585	shot by the author	1280 × 960	180 in total
Out-of-focus [25]	30	150	1	5/150	shot by the author	720 × 480	26
Our ZPHD	45	900	20	N.A./900	shot by the author	4000 × 3000	10 experts

**Table 3:** Overall prediction performance on our zoom photo database.

Methods	SROCC	KROCC	PLCC
NIQE [32]	0.8493	0.6863	0.8490
SNP-NIQE [33]	0.7216	0.3754	0.6932
IL-NIQE [65]	0.5022	0.3034	0.5257
NPQI [74]	0.7247	0.5506	0.7106
LPSI [75]	0.7538	0.6620	0.7435
QAC [76]	0.6892	0.5831	0.6431
BIQI [77]	0.7576	0.5419	0.7344
BRISQUE [31]	0.6385	0.4530	0.6700
BLIINDS-II [64]	0.5265	0.3031	0.5134
M3 [78]	0.4078	0.2263	0.4530
JNB [13]	0.7580	0.6678	0.7699
CPBD [18]	0.8305	0.6713	0.8017
FISH [15]	0.7662	0.6076	0.7423
LPC [19]	0.8567	0.7046	0.8436
SPARISH [23]	0.8456	0.6871	0.8328
S3 [17]	0.7962	0.6153	0.7829
S3-III [27]	0.8114	0.6347	0.8163
The proposed metric	**0.9216**	**0.7908**	**0.9129**

## Data Availability

The zoom photo database is available at https://drive.google.com/file/d/1c9rflGXu7_rd6Va_1Sv9GL9lQByJeZXf (accessed on 20 March 2023).

## Abbreviations

The following abbreviations are used in this manuscript:

CMOS	Complementary Metal Oxide Semiconductor
DSLR	Digital Single-Lens Reflex
ISP	Image Signal Processor
IQA	Image Quality Assessment
PDAF	Phase-Detection Auto Focus
MOS	Mean Opinion Score
RR-IQA	Reduced-Reference Image Quality Assessment
NR-IQA	No-Reference Image Quality Assessment
HDR	High Dynamic Range
SROCC	Spearman Rank Order Correlation Coefficient
KROCC	Kendall Rank Order Correlation Coefficient
PLCC	Pearson Linear Correlation Coefficient
VQEG	Video Quality Experts Group
